# Relationship Between Drop Jump Training–Induced Changes in Passive Plantar Flexor Stiffness and Explosive Performance

**DOI:** 10.3389/fphys.2021.777268

**Published:** 2021-11-18

**Authors:** Ryosuke Ando, Shinya Sato, Naoya Hirata, Hiroki Tanimoto, Naoto Imaizumi, Yasuhiro Suzuki, Kosuke Hirata, Ryota Akagi

**Affiliations:** ^1^Department of Sports Research, Japan Institute of Sports Sciences (JISS), Tokyo, Japan; ^2^Graduate School of Engineering and Science, Shibaura Institute of Technology, Saitama, Japan; ^3^Graduate School of Health Management, Keio University, Fujisawa, Japan; ^4^College of System Engineering and Science, Shibaura Institute of Technology, Saitama, Japan; ^5^Center for General Education, Tokyo Keizai University, Tokyo, Japan; ^6^Japan Society for the Promotion of Science, Tokyo, Japan; ^7^Faculty of Sport Sciences, Waseda University, Saitama, Japan

**Keywords:** passive muscle stiffness, gastrocnemius, soleus, rate of torque development, drop jump, elastography

## Abstract

Passive muscle stiffness is positively associated with explosive performance. Drop jump training may be a strategy to increase passive muscle stiffness in the lower limb muscles. Therefore, the purpose of this study was to examine the effect of 8-week drop jump training on the passive stiffness in the plantar flexor muscles and the association between training-induced changes in passive muscle stiffness and explosive performance. This study was a randomized controlled trial. Twenty-four healthy young men were divided into two groups, control and training. The participants in the training group performed drop jumps (five sets of 20 repetitions each) 3days per week for 8weeks. As an index of passive muscle stiffness, the shear moduli of the medial gastrocnemius and soleus were measured by shear wave elastography before and after the intervention. The participants performed maximal voluntary isometric plantar flexion at an ankle joint angle of 0° and maximal drop jumps from a 15cm high box. The rate of torque development during isometric contraction was calculated. The shear modulus of the medial gastrocnemius decreased for the training group (before: 13.5±2.1kPa, after: 10.6±2.1kPa); however, such a reduction was not observed in the control group. There was no significant group (control and training groups)×time (before and after the intervention) interaction for the shear modulus of the soleus. The drop jump performance for the training group improved, while the rate of torque development did not change. Relative changes in these measurements were not correlated with each other in the training group. These results suggest that drop jump training decreases the passive stiffness in the medial gastrocnemius, and training-induced improvement in explosive performance cannot be attributed to change in passive muscle stiffness.

## Introduction

Passive muscle stiffness (muscle stiffness at rest) could be an important determinant in sports performance because it has been found to be associated with explosive performance (Takahashi et al., 2018; Ando and Suzuki, 2019; Miyamoto et al., 2019; Ando et al., 2021). For example, we previously showed positive correlations of passive medial gastrocnemius (MG) stiffness with the rate of torque development (RTD) during maximal isometric plantar flexion (Ando and Suzuki, 2019) and with drop jump performance (Ando et al., 2021). In addition, Miyamoto et al. (2019) showed that passive vastus lateralis stiffness positively related to the sprinting ability of sprinters. These results suggest that passive muscle stiffness is not a negligible influencing factor for considering the stretch-shortening cycle in the muscle-tendon unit, even though it is generally known that tendon stiffness is the major factor.

To the best of our knowledge, a training regimen that increases passive muscle stiffness has not yet been well developed. In an animal study, an increase in the passive stiffness of the extensor digitorum longus and rectus femoris was demonstrated after 15-week jump training (Ducomps et al., 2003). In a human study, increased passive stiffness was demonstrated in type IIa/IIx fibers of the vastus lateralis after 8weeks of various jump trainings (Malisoux et al., 2006). Fouré et al. (2009, 2011) also indicated a possibility that 8weeks of jump training increases the passive stiffness in the gastrocnemius of humans. However, they regarded the ankle joint stiffness calculated from the passive plantar flexor torque during dorsiflexion as passive muscle stiffness. Because joint stiffness is affected not only by the muscle but also the joint capsule, tendon, skin, and so on (Johns and Wright, 1962), the effect of long-term jump training on passive stiffness in the plantar flexor muscles is still unclear. Recently, ultrasound shear wave elastography was used to directly measure the longitudinal elasticity of muscle *in vivo*, and the results showed similar relationships between passive muscle stiffness and explosive performance as described above (Ando and Suzuki, 2019; Miyamoto et al., 2019; Ando et al., 2021). Therefore, ultrasound shear wave elastography must be employed to clarify the effect of jump training on muscle stiffness.

Furthermore, previous studies (Malisoux et al., 2006; Fouré et al., 2009, 2011) have not focused on the association between training-induced changes in passive muscle stiffness and explosive performance, even though passive muscle stiffness is considered to influence explosive performance. We clarified that the passive stiffness of the MG, not soleus (SOL), is positively related to RTD during isometric contraction (Ando and Suzuki, 2019) and drop jump performance (Ando et al., 2021). Thus, increased passive MG stiffness could be advantageous to explosive performance. In many types of jump training, drop jumping imposes considerable stress on the plantar flexor muscles because greater plantar flexor torque is produced in the contact phase of a drop jump than in the contact phase of a counter-movement jump (Bobbert et al., 1987a). Considering the necessity of sufficient mechanical stress for changing the tissue mechanical property (Kjaer, 2004), long-term drop jump training can be one of the best interventions to stiffen the passive plantar flexor muscles, thus enhancing the explosive performance.

Therefore, the purpose of this study was to examine the effect of 8-week drop jump training on the passive stiffness in the plantar flexor muscles *via* ultrasound shear wave elastography and to determine the relationship between training-induced changes in passive muscle stiffness and explosive performance. We hypothesized that the passive stiffness in the plantar flexor muscles increased after 8-week drop jump training and training-induced change in passive MG stiffness associated with change in explosive performance.

## Materials and Methods

### Participants

Twenty-four healthy young men participated in an open, parallel group, randomized controlled intervention study. This number was determined by a sample size estimation using the data of a previous study that examined the effect of 8weeks of jump training on the stiffness of plantar flexor muscles (Fouré et al., 2009). Based on an *α*-level of 0.05 and a power (1−*β*) of 0.80, and an expected 33% change in passive muscle stiffness, the analysis indicated that at least 11 participants were required in each group. The intervention period of 8weeks was determined based on previous studies that suggested increased passive muscle stiffness in the vastus lateralis and gastrocnemius after 8weeks of jump training (Malisoux et al., 2006; Fouré et al., 2009). Participants were excluded from the present study if they were particularly well-trained in running, jumping, ball games, or any other form of athletics that could potentially impact the passive stiffness of their plantar flexor muscles or if they had any history of orthopedic surgery on the lower limb. They were randomly divided into a control group (*n*=12, age: 22±1years, height: 170.3±3.7cm, body mass: 64.8±12.6kg) and training group (*n*=12, age: 22±1years, height: 176.1±7.5cm, body mass: 66.9±5.6kg) using block randomization through a random number table. Before proceeding with the experiment, the purpose of the study, its procedures, and associated risks were explained to all the participants and written informed consent was obtained from them. The experimental protocols were approved by the ethics committees of the Japan Institute of Sports Sciences (No. 027) and the Shibaura Institute of Technology (No. 18-009). This study was conducted in accordance with the Declaration of Helsinki.

### Experimental Design

The participants in the training group underwent 8-week drop jump training and maintained their habitual daily physical activity. The participants in the control group were asked to refrain from any resistance or plyometric training during the control period. Before and after the 8-week control and training periods, the muscle shear moduli of the MG and SOL, isometric plantar flexor torque, RTD, electromechanical delay (EMD), rate of electromyogram rise (RER), and drop jump performance were determined. Because plantar flexor muscles produce greater torque than knee and hip extensor muscles during the drop jump (Bobbert et al., 1987a), we evaluated the functional and mechanical properties of plantar flexor muscles. All the participants visited the laboratory at least 1week prior to testing for a familiarization trial.

### Drop Jump Training

The participants in the training group performed drop jumps 3days per week for 8weeks. The training consisted of five sets of 20 drop jumps each with a 1-min rest between the sets. The participants dropped from a 15cm high box in the first 4weeks and a 30cm high box in the next 4weeks. They were instructed to jump as high as possible with a short contact time for each jump. While jumping, they kept their hands on their hips. They were instructed to avoid deep flexion of the knee and hip joints to ensure a short contact time, and this was practiced during a familiarization trial (described later).

### Muscle Shear Modulus

The shear wave propagation speed of the muscle was measured using ultrasound shear wave elastography (ACUSON S2000; Siemens Medical Solutions, United States), which was used to calculate the shear modulus (as described below) and thereby evaluate the passive muscle stiffness. The participants lay prone on an examination bed with their knees fully extended and their right foot secured to the footplate of an electrical dynamometer (CON-TREX MJ; Physiomed, Germany). The ankle joint was fixed at 0° (neutral position, 90° between the foot and tibia), and the participants were asked to relax their leg during measurement. The shear wave propagation speed through the MG was measured at the proximal 30% position of the leg length from the popliteal crease to the lateral malleolus and at the 40% position of the girth from the boundary between the MG and tibia to that between the MG and lateral gastrocnemius. The shear wave propagation speed through the SOL, on the other hand, was measured at the proximal 30% position of the leg length and at the 20% position of the girth from the boundary between the MG and lateral gastrocnemius to that between the MG and tibia. After determining the measurement sites, the ankle joint was plantar flexed to 40° from the neutral position. This position was sustained with relaxation for 5min to eliminate the effects of static stretching at an ankle joint angle of 0°. The ankle joint was then returned to the neutral position; furthermore, the shear wave propagation speed was immediately measured thrice for each muscle in a random order. A linear array probe (9 L4 Transducer, 4–9MHz; Siemens Medical Solutions) was longitudinally placed at each measurement site with sufficient water-soluble transmission gel, and its direction was adjusted to the orientation of muscle fascicles. The coefficient of variance for the three measurements was 3.6±2.0% for the MG and 2.7±1.7% for the SOL. To ensure the relaxation of muscle during scanning, electromyogram (EMG, described below) was recorded. The root-mean-square (RMS) of the EMG signals during scanning normalized by that during maximal voluntary isometric contraction (MVC) was 1.9±1.3% for the MG and 2.1±1.9% for the SOL.

**Figure 1:**
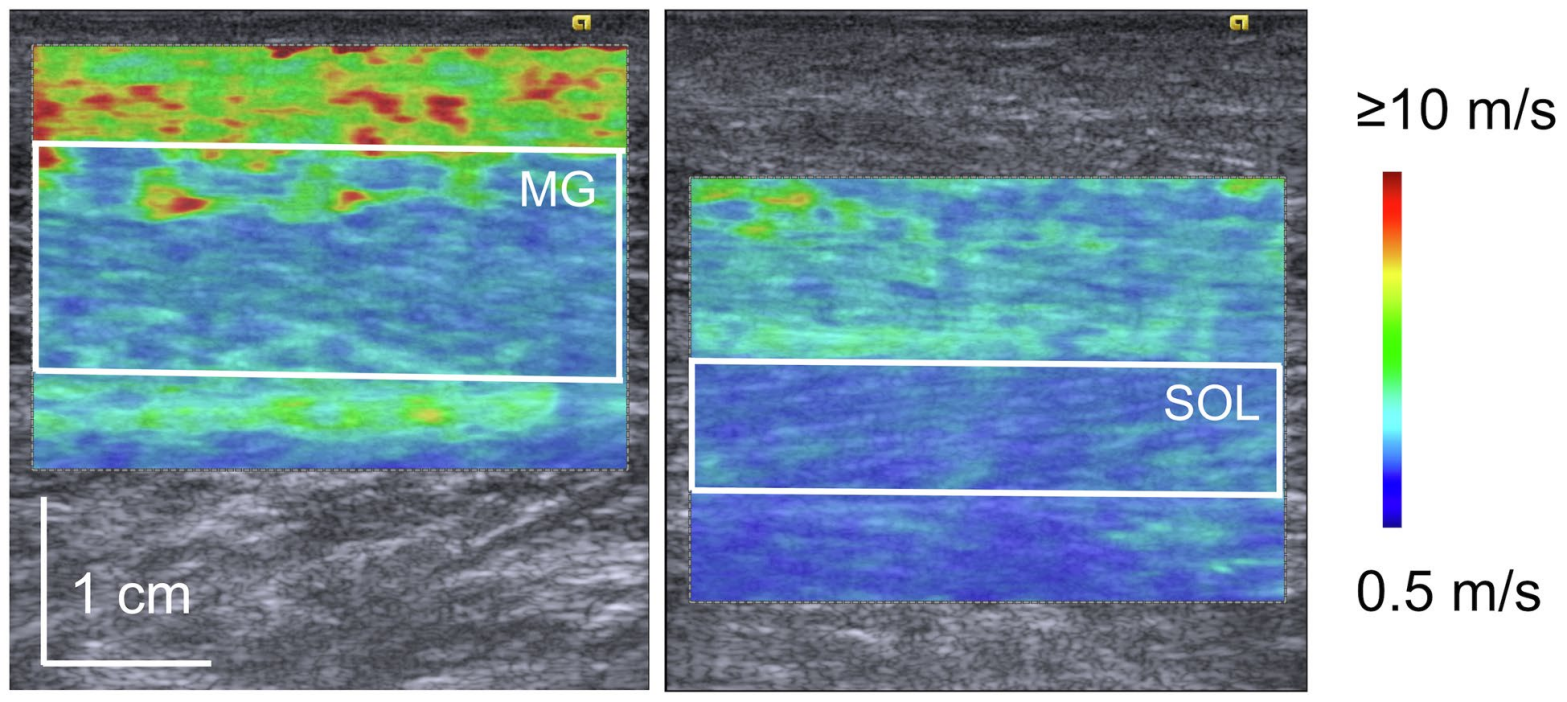
Representative elastography images of medial gastrocnemius (MG) and soleus (SOL). The white square in the image represents the region of interest for shear wave elastography. Blue color indicates the lower speed of shear wave propagation while red indicates the higher speed of shear wave propagation.

The shear modulus was calculated using elastography images according to a procedure described in a previous paper (Hirata et al., 2020). The elastography images were exported in the Digital Imaging and Communications in Medicine (DICOM) format from the ultrasound apparatus. The region of interest on the elastography image color map was selected to be as large as possible (Figure 1) using an image processing software (ImageJ; NIH, United States). Tissues other than the target muscle (subcutaneous fat, aponeurosis, fascia, etc.) were not included in the selected area. Then, the red-green-blue (RGB) value of each pixel within the region of interest was converted into shear wave speed according to the RGB value–shear wave speed relationship estimated using the color scale displayed on the elastography image. The shear modulus of each pixel was calculated by multiplying the shear wave speed and tissue density (Hug et al., 2015). In the present study, the muscle density was assumed to be 1,000 kg/m^3^ (Freitas et al., 2015; Hug et al., 2015). Thereafter, the muscle shear modulus of each elastography image was calculated by averaging the shear moduli of all pixels in the region of interest.

### Plantar Flexion Task

The participants performed isometric plantar flexion at an ankle joint angle of 0° following the ultrasound shear wave elastography measurements. They performed several submaximal isometric contractions as a warm-up exercise. Two MVCs were performed for 4s, and an additional trial was performed if the peak torque values differed by >10% between two trials. A rest of 1min was allowed between trials. Subsequently, the participants performed 10 maximal isometric plantar flexions as fast and hard as possible and sustained maximal effort for approximately 1s with a rest of 30s between flexions.

Signals from the dynamometer, which were recorded on a personal computer using the LabChart v8.1.5 software (ADInstruments, Australia), were sampled at 2kHz using an analog-to-digital converter (PowerLab; ADInstruments). The torque signal was sampled and averaged over 1s during the sustained phase of an MVC to calculate the MVC torque. Out of the two values obtained, the higher value was used as the representative value for further analyses. The normalized RTD was calculated based on previous studies (Aagaard et al., 2002; Ema et al., 2017; Ando and Suzuki, 2019). First, the onset of plantar flexion was determined as the point at which the torque exceeded the baseline by 2.5% of the MVC torque. To eliminate the effect of counter movement on RTD values, trials in which >0.5Nm of dorsiflexion torque was observed before the onset were excluded in accordance with observations made in previous studies (Tillin et al., 2010; Balshaw et al., 2016). The RTD was then calculated as the slope of the time–torque curve over time intervals of 0–100 and 0–200ms from the onset of plantar flexion. These RTD values were normalized by the MVC torque to exclude the effect of muscle strength. The average of the highest three RTD values was used for further analyses.

### Electromyogram Recording

Surface EMG signals were acquired from the MG and SOL during the ultrasound shear wave elastography measurements and the isometric plantar flexion task. The measurement site of the EMG signals for the MG was located at the proximal 30% position of the leg length. The electrode placement for the SOL was <5cm from the distal 40% position of the leg length, but interindividual variations were found because of the variability in the superficial region of the SOL. At these points, fascicle longitudinal directions were confirmed by B-mode ultrasonography. A single differential electrode (DE-2.1 Sensor; Delsys, United States) was used, which had an interelectrode distance of 1cm, a contact sensor composed of two silver bars (0.1×1cm each), an input impedance of >10^15^Ω per 0.2pF, and a common rejection ratio of 90dB. The sensor preamplifier and main amplifier units (Bagnoli-8; Delsys) were set to 10- and 100-fold gains, respectively, which resulted in a 1,000-fold amplification of the original EMG signal and a frequency response of 20±5 to 450±50Hz. The signals from the EMG system were sampled at 2kHz using an analog-to-digital converter and synchronized with the torque data on a personal computer using LabChart v8.1.5 software.

The EMD and RER were calculated according to procedures described in a previous study (Ema et al., 2017). Briefly, EMG signals were smoothed using a moving RMS window of 50ms during the RTD test. The onset of muscle activity was set at mean +3 standard deviations (SDs) of the EMG RMS value from the baseline (mean and SD were calculated at rest over a time window of 200ms). The EMD was determined as the difference in time between the onset of plantar flexor torque and MG activation, which was closely related to tendon stiffness (Waugh et al., 2013). The RER of the MG (RER_MG_) and SOL (RER_SOL_) was determined as the slope of the smoothed time–EMG curve from EMG onset to 50ms. The reason for selecting a time interval of 50ms was based on the association between changes in the rate of force development and RER at a time interval of 50ms after 10-week isokinetic training (Blazevich et al., 2008).

### Drop Jump Test

The participants dropped from a 15cm high box, landed on a jump analysis mat (DKH Co. Ltd., Japan), immediately jumped as high as possible, and landed on the mat again. The drop jump test was conducted using a 15cm high box because relative contribution of the plantar flexors torque to drop jump was similar among different box heights in the previous study (Bobbert et al., 1987b). The instructions for the drop jump were the same as those for the drop jump training (described above). The signals from the mat, which were recorded on a personal computer using the Multi Jump Tester software (DKH Co. Ltd.), were sampled using an analog-to-digital converter. The jumping height was calculated using the following equation (Yoon et al., 2007; Miura et al., 2010):


$$\mathrm{Jumping height}=1/8\times g\times t^{2}$$

where *g* and *t* are the gravitational acceleration (9.81m/s^2^) and flight time, respectively. The reactive strength index (RSI) was calculated by dividing the jump height (m) by the contact time (s). Drop jumps were performed twice. The highest RSI and the associated jump height and contact time were used for further analyses.

### Statistical Analyses

Statistical analyses were performed using IBM SPSS Statistics software (version 24.0; IBM, United States). Normality was tested using the Shapiro–Wilk test, which did not indicate normality for the data of some parameters. In the present study, a two-way repeated-measures analysis of variance (ANOVA) was required to examine the group (control and training groups) and time (before and after the intervention) interaction. Therefore, all data were log transformed, which were then confirmed as the normal distribution. The two-way ANOVA with repeated measures (group and time) was used to evaluate changes in the shear moduli of the MG and SOL, MVC torque, RTD at each time interval, EMD, RER_MG_, RER_SOL_, RSI, jump height, and contact time before and after the intervention. When an interaction was found, an unpaired/paired *t*-test was performed for group/time comparison with Bonferroni’s correction. For the interaction and main effect of the two-way ANOVA, *η*^2^ was calculated as an index of the effect size. Furthermore, Cohen’s *d* was appropriately calculated as an index of the effect size for an unpaired/paired *t*-test. The values of *η*^2^ or *d* were interpreted as *η*^2^<0.01 or *d*<0.20 for trivial effects, 0.01≤*η*^2^<0.06 or 0.20≤*d*<0.50 for small effects, 0.06≤*η*^2^<0.14 or 0.50≤*d*<0.80 for medium effects, and 0.14≤*η*^2^ or 0.80≤*d* for large effects (Cohen, 1988). The relationship of the relative changes in shear moduli of the MG and SOL with RTD_100_, RTD_200_, and RSI from the baseline to 8weeks later was examined for the training group using Pearson’s product-moment correlation coefficient. All the data were presented as mean±SD. The level of significance was set at *p*<0.05 for all the analyses.

## Results

The data of the shear moduli of the MG and SOL, MVC torque, RTD, EMD, RER of the MG and SOL, drop jump performance, ANOVA results, and effect size results are presented in Table 1. A significant group×time interaction was found for the MG shear modulus. The MG shear modulus increased from the baseline to 8weeks later for the control group (*p*=0.001, *d*=0.86), whereas it decreased for the training group (*p*=0.001, *d*=1.38). The MG shear modulus was lower for the training group than that for the control group after the intervention (*p*<0.001, *d*=1.63). No significant group×time interaction was found for the SOL shear modulus, and no main effect of the group on the SOL shear modulus was observed. A significant main effect of time on the SOL shear modulus was found, indicating a decreased shear modulus in both groups. A significant main effect of the group on the MVC torque was observed, but no significant group×time interaction or main effect of time on the MVC torque was found. No significant interactions or main effects were found for the RTD, EMD, and RER of the MG and SOL. A significant group×time interaction was found for the RSI. *Post-hoc* tests indicated that the RSI increased from the baseline to 8weeks later for the training group (*p*<0.001, *d*=1.15), whereas it did not change for the control group (*p*=0.278, *d*=0.14). The RSI was greater for the training group than the control group after the intervention (*p*=0.001, *d*=1.40). A main effect of time on the jump height was found, indicating an increased jump height for the control group and training group. No significant group×time interaction or main effect of group on the jump height was found. A significant group×time interaction was observed for the contact time. Multiple comparisons indicated that the contact time decreased from the baseline to 8weeks later for the training group (*p*=0.001, *d*=1.08), whereas it did not change for the control group (*p*=0.625, *d*=0.20). The contact time was lower for the training group than the control group after the intervention (*p*=0.004, *d*=1.35).

**Table 1:** Shear modulus, MVC torque, RTD, EMD, RER, RSI, jump height, and contact time for control and training groups.

	Control		Training		ANOVA	Effect size
	Before	After	Before	After		
MG shear modulus (kPa)	12.7±3.6	16.1±4.3^*^	13.5±2.1	10.6±2.1^*^^,^^†^	Group: *p*=0.071, time: *p*=0.985, group×time: *p*<0.001	Group: *η*^2^=0.19, time: *η*^2^<0.01, group×time: *η*^2^=0.46
SOL shear modulus (kPa)	5.3±2.2	4.9±0.7	5.8±1.1	4.7±0.8	Group: *p*=0.597, time: *p*=0.025, group×time: *p*=0.168	Group: *η*^2^=0.01, time: *η*^2^=0.19, group×time: *η*^2^=0.07
MVC torque (Nm)	118±21	115±15	122±21	134±16	Group: *p*=0.049, time: *p*=0.291, group×time: *p*=0.152	Group: *η*^2^=0.21, time: *η*^2^=0.04, group×time: *η*^2^=0.08
RTD_100_ (%MVC/s)	353±105	333±44	407±118	395±113	Group: *p*=0.215, time: *p*=0.529, group×time: *p*=0.986	Group: *η*^2^=0.33, time: *η*^2^=0.02, group×time: *η*^2^<0.01
RTD_200_ (%MVC/s)	305±51	311±23	340±67	335±56	Group: *p*=0.202, time: *p*=0.724, group×time: *p*=0.497	Group: *η*^2^=0.27, time: *η*^2^<0.01, group×time: *η*^2^=0.02
EMD (ms)	78±15	80±15	69±11	69±14	Group: *p*=0.083, time: *p*=0.799, group × time: *p*=0.672	Group: *η*^2^=0.43, time: *η*^2^<0.01, group×time: *η*^2^<0.01
RER_MG_ (mV/s)	0.84±0.37	0.72±0.42	1.05±0.68	0.94±0.61	Group: *p*=0.399, time: *p*=0.098, group×time: *p*=0.538	Group: *η*^2^=0.14, time: *η*^2^=0.10, group×time: *η*^2^<0.01
RER_SOL_ (mV/s)	1.25±0.90	1.14±0.65	1.85±1.23	1.77±1.26	Group: *p*=0.532, time: *p*=0.430, group×time: *p*=0.276	Group: *η*^2^=0.09, time: *η*^2^=0.03, group×time: *η*^2^<0.01
RSI (m/s)	0.88±0.42	0.93±0.27	0.96±0.42	1.48±0.47^*^^,^^†^	Group: *p*=0.090, time: *p*<0.001, group×time: *p*=0.007	Group: *η*^2^=0.23, time: *η*^2^=0.31, group×time: *η*^2^=0.13
Jump height (cm)	17.9±7.3	19.1±5.7	19.8±7.8	25.8±6.8	Group: *p*=0.173, time: *p*=0.005, group×time: *p*=0.081	Group: *η*^2^=0.19, time: *η*^2^=0.23, group×time: *η*^2^<0.08
Contact time (ms)	214±36	208±22	213±41	178±23^*^^,^^†^	Group: *p*=0.124, time: *p*=0.006, group×time: *p*=0.030	Group: *η*^2^=0.15, time: *η*^2^=0.21, group×time: *η*^2^=0.13

Values are presented as mean±standard deviation. ANOVA, analysis of variance; MVC, maximal voluntary contraction; MG, medial gastrocnemius; SOL, soleus; RTD, rate of torque development; EMD, electromechanical delay; RER, rate of electromyography rise; RSI, reactive strength index.

* *p*<0.05 vs. pre by *post-hoc* test.

† *p*<0.05 vs. control by *post-hoc* test.

**Figure 2:**
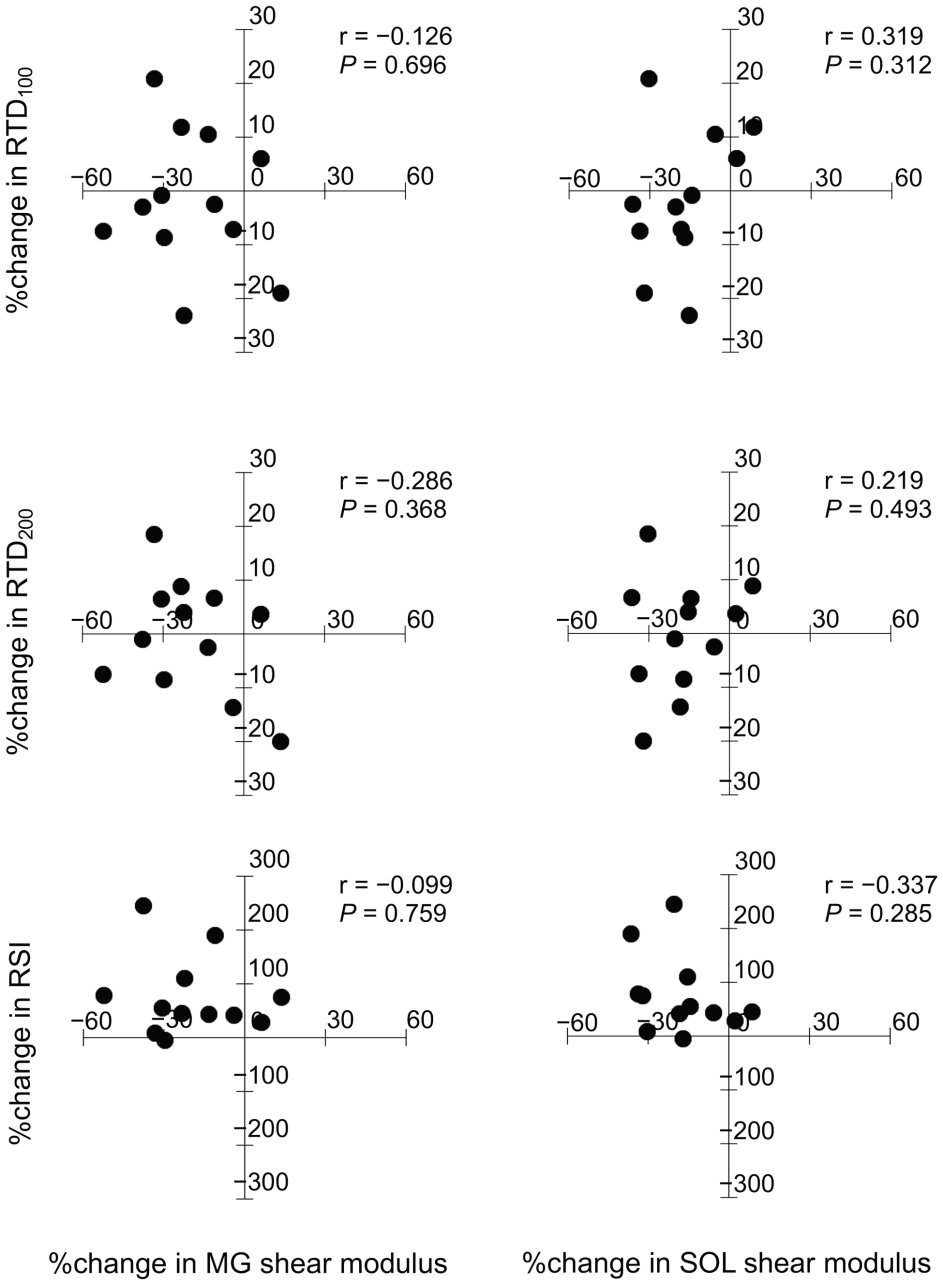
Relationships between the relative changes in shear moduli of the medial gastrocnemius (MG) and soleus (SOL) with rate of torque development (RTD_100_, RTD_200_), and reactive strength index (RSI).

Figure 2 shows relationships between the relative changes in shear moduli of the MG and SOL with RTD_100_, RTD_200_, and RSI from the baseline to after the intervention for the training group. Shear moduli of the MG and SOL did not significantly correlate with RTD_100_, RTD_200_, and RSI.

## Discussion

This study examined the effect of 8-week drop jump training on the passive stiffness in the plantar flexor muscles and the relationship between training-induced changes in passive muscle stiffness and explosive performance. In the training group, after the intervention, the MG shear modulus decreased, the RTD did not change, and the drop jump performance improved. Furthermore, relative changes in the shear moduli of the MG and SOL did not correlate with those in the RTD and RSI in the training group. These results suggest that 8-week drop jump training decreases the passive MG stiffness; however, improvement in explosive performance cannot be attributed to change in passive muscle stiffness.

Contrary to our hypothesis, the MG shear modulus for the training group decreased with a large effect size after 8weeks. This could be due to lower drop jump performance-related fascicle behavior in the contact phase during drop jumping in training periods. The performance of reactive jumps such as drop jumps or rebound jumps was dramatically lower in the present study than in previous studies (Fouré et al., 2009, 2011; Laurent et al., 2020). The fascicle lengthening in the participants was speculated in the contact phase during drop jump because the MG fascicles behaved isometrically or shortened in the contact phase during drop jumping (Ishikawa et al., 2005), and the magnitude of fascicle shortening was greater in high-performance individuals (Hoffrén et al., 2007). Blazevich (2019) suggested that long-term training, including eccentric muscular contraction, decreases passive muscle stiffness. Taken together, drop jump training might impose lengthening of the MG fascicles in most of our participants, which would lead to a decrease in the MG shear modulus. Because fascicle lengthening in the contact phase during drop jump was not proven by ultrasound imaging or kinematic analysis, further studies are warranted in the future.

Because the MG shear modulus decreased in the present study, a decrease in the RTD must be expected based on the positive correlation between the MG shear modulus and RTD (Ando and Suzuki, 2019). However, no significant changes were observed in RTD_100_ and RTD_200_, and no significant correlations were found between the relative changes in the MG shear modulus and RTD_100_ or RTD_200_. These results were attributed to the possibility that the effect of changes in passive muscle stiffness on the RTD was masked by changes in muscle activation. Muscle activation, as assessed by surface EMG, is a major factor influencing the rate of force development (Maffiuletti et al., 2016). A strong correlation was found between the relative changes in RER_MG_ and RTD_100_ in the training group (*r*=0.735). We previously found moderate correlations between the MG shear modulus and RTD in a cross-sectional study (*r*=0.460–0.496; Ando and Suzuki, 2019). Therefore, influence of the change in the MG shear modulus was relatively low compared with that in muscle activation.

We expected an association between training-induced changes in the MG shear modulus and the RSI because of a positive relationship between the MG shear modulus and drop jump performance (Ando et al., 2021). However, the drop jump performance for the training group improved after the intervention, whereas the MG shear modulus decreased. Therefore, other determining factors such as muscle strength, mechanical properties of tendons, muscle fiber type, and neural factors could have contributed to the increase in the drop jump performance in the present study. Regarding muscle strength, no significant group×time interaction was found for the MVC torque. In addition, the EMD, which is an index of tendon stiffness (Waugh et al., 2013), did not change before and after the intervention in both the groups. Subsequently, the muscle fiber type composition was assumed to not change during the 8-week drop jump training. This is because no change in the fiber type composition was observed after an 8-week ballistic resistance exercise (Winchester et al., 2008), which would cause higher training stress than drop jump training. Thus, changes in neural factors would improve the drop jump performance in the present study. Hoffrén et al. (2007) showed that active joint stiffness was related to plantar flexor muscle activation in the early contact phase (i.e., braking phase) during drop jumping. Furthermore, they indicated an association between active joint stiffness and drop jump performance. Therefore, we believe that increased active joint stiffness due to muscle activity (i.e., a neural factor) strongly affects the improvement in the drop jump performance in the training group. Thus, the effect of decreased MG shear modulus might disappear.

Previous studies have reported inconsistent results regarding the effect of jump training, including drop jumping, on Achilles tendon stiffness. One study reported that Achilles tendon stiffness increased after long-term jump training (Laurent et al., 2020), whereas others found that it decreased or did not change (Kubo et al., 2007; Fouré et al., 2009, 2011; Houghton et al., 2013). In the present study, no significant change was found in the EMD, which was closely related to tendon stiffness (Waugh et al., 2013), before and after the 8-week drop jump training. This result indicates a possibility that Achilles tendon stiffness did not change. Reactive jump performance has been shown to be related to Achilles tendon stiffness (Abdelsattar et al., 2018; Laurent et al., 2020). However, drop jump performance improved without a change in the EMD in the present study. Because our participants showed lower drop jump performance (especially jump height), it is speculated that the first step toward improving drop jump performance is to increase active joint stiffness due to muscle activity and then increase Achilles tendon stiffness or other factors.

There were limitations in the present study. First, the shear moduli of the MG and SOL changed significantly in the control group after the 8-week control period. These results may be partly due to inter-days difference for measurement of the shear modulus. However, the effect size of the MG shear modulus between before and after the intervention was greater for the training group (*d*=1.38) than for the control group (*d*=0.86). Therefore, decreased MG shear modulus after the 8-week drop jump training would be meaningful. Second, in the present study, no muscular contraction was performed before the ultrasound scanning, although Stubbs et al. (2018) suggested that contraction at short lengths eliminates the history dependence of the muscle slack length. However, the procedures for ultrasound scanning before and after the intervention were the same for all the participants (e.g., relaxation of the muscle at the slacked length for 5min before ultrasound scanning). Therefore, lack of muscular contraction would not have affected the interpretation of our results. Third, no measurements of kinetics and kinematics of the lower limbs were taken during drop jumping before and after the 8-week training program. Therefore, the improvement in the drop jump performance in the training group may be attributed to increased torque by the ankle, knee, and hip joints. The ankle joint produced more torque than the knee and hip joints in the contact phase during drop jumping (Bobbert et al., 1987a), resulting in higher stress imposed on the plantar flexor muscles than the knee and hip extensor muscles during the training period. In the present study, no significant increase was found in the MVC torque in the plantar flexor muscles for the training group (Table 1). Thus, no increase in the muscle strength of the knee and hip extensors was expected. Fourth, the height of the box was increased up to 30cm during the intervention in the present study. Bobbert et al. (1987b) indicated that the torque produced by the plantar flexors was greater using a higher box (20–60cm). Therefore, further training effect might be gained if the height of box was increased further during the intervention. However, because participants in the present study could not be tested for higher physical activity before recruiting for the experiment and performed poorly in the drop jump test, injury risk during training needed to be avoided. Further studies in this regard are warranted in the future. Finally, an 8-week training program was adopted in the present study. A longer training period is generally considered to induce greater morphological and functional changes. A longer intervention (e.g., 12weeks) could improve RTD and indicate a significant group×time interaction for the SOL shear modulus. Furthermore, the relative changes in the shear moduli may be related to those in RTD and RSI.

In conclusion, the 8-week drop jump training decreased the MG shear modulus, but this was not related with any change in RSI. These results suggest that drop jump training decreases the passive stiffness in the MG, and training-induced improvement in explosive performance cannot be attributed to change in passive muscle stiffness.

## Data Availability Statement

The raw data supporting the conclusions of this article will be made available by the authors, without undue reservation.

## Ethics Statement

The studies involving human participants were reviewed and approved by Japan Institute of Sports Sciences and Shibaura Institute of Technology. The patients/participants provided their written informed consent to participate in this study.

## Author Contributions

RA, SS, YS, KH, and RA: conceived and designed the experiments. RA, SS, NH, HT, NI, and KH: performed experiment. RA and KH: analyzed data. RA: drafted manuscript and prepared tables and figures. All authors interpreted the results of the research, edited, critically revised, and approved the final version of the manuscript, and have agreed to be accountable for all aspects of the work related to its accuracy and integrity.

## Funding

This study was supported in part by JSPS KAKENHI grants to RAn (grant number 18K17813) and RAk (grant numbers 16H05918 and 17KK0174).

## Conflict of Interest

The authors declare that the research was conducted in the absence of any commercial or financial relationships that could be construed as a potential conflict of interest.

## Publisher’s Note

All claims expressed in this article are solely those of the authors and do not necessarily represent those of their affiliated organizations, or those of the publisher, the editors and the reviewers. Any product that may be evaluated in this article, or claim that may be made by its manufacturer, is not guaranteed or endorsed by the publisher.
